# Beyond Bone Mineral Density: A New Dual X-Ray Absorptiometry Index of Bone Strength to Predict Fragility Fractures, the Bone Strain Index

**DOI:** 10.3389/fmed.2020.590139

**Published:** 2021-01-15

**Authors:** Fabio Massimo Ulivieri, Luca Rinaudo

**Affiliations:** ^1^Fondazione Istituto di Ricovero e Cura a Carattere Scientifico (IRCCS) Ca' Granda Ospedale Maggiore Policlinico, Unità Operativa (UO) Medicina Nucleare, Milan, Italy; ^2^Technologic Srl, Turin, Italy

**Keywords:** DXA (dual x-ray absorptiometry), TBS (trabecular bone score), BSI (bone strain index), BMD (bone mineral density), osteoporosis, HSA (hip structural analysis)

## Abstract

For a proper assessment of osteoporotic fragility fracture prediction, all aspects regarding bone mineral density, bone texture, geometry and information about strength are necessary, particularly in endocrinological and rheumatological diseases, where bone quality impairment is relevant. Data regarding bone quantity (density) and, partially, bone quality (structure and geometry) are obtained by the gold standard method of dual X-ray absorptiometry (DXA). Data about bone strength are not yet readily available. To evaluate bone resistance to strain, a new DXA-derived index based on the Finite Element Analysis (FEA) of a greyscale of density distribution measured on spine and femoral scan, namely Bone Strain Index (BSI), has recently been developed. Bone Strain Index includes local information on density distribution, bone geometry and loadings and it differs from bone mineral density (BMD) and other variables of bone quality like trabecular bone score (TBS), which are all based on the quantification of bone mass and distribution averaged over the scanned region. This state of the art review illustrates the methodology of BSI calculation, the findings of its in reproducibility and the preliminary data about its capability to predict fragility fracture and to monitor the follow up of the pharmacological treatment for osteoporosis.

## Introduction

Rheumatological diseases are chronic inflammatory illnesses characterised by local and systemic multifactorial bone loss (1). Systemic inflammation, with the secretion of pro-inflammatory cytokines, and steroid drugs, frequently prescribed for the therapy, play a crucial role in the local and systemic pathogenesis of bone loss and its common manifestations, like reduced bone mass, osteopenia and osteoporosis (2, 3).

This classification of bone derangement is based on the commonly used diagnostic dual X-ray absorptiometry (DXA) method to assess bone quantity (bone mineral density, BMD), bone quality (trabecular bone score, TBS) and bone geometry (hip structural analysis, HSA) (4–6). Osteoporosis is present when BMD, expressed in standard deviation from a healthy young population, is ≤ –2.5 for postmenopausal women and for over 50 years old men, while osteopenia is defined as a T-score ≤ –1.0. For the other ages of men and premenopausal females, BMD is expressed as a standard deviation from age- and sex-matched population with the cutoff set at ≤ –2.0 (7).

Even though DXA devices are the most widespread, other methods can be used to investigate bone status. QCT (8) and Quantitative UltraSound (QUS) (9) have been applied for several years in osteoporosis management, and more recently a radiofrequency echographic technique based on the analysis of raw ultrasound signals has been proposed (10–12).

Bone involvement is a well-known implication in Rheumatoid Arthritis (RA), where systemic bone loss is one of the most common comorbidities, that starts early in disease development, even before the clinical onset of this rheumatological disease (1).

The skeletal sites affected are mainly those with cortical bone, like femoral neck and distal radius and those with prevalent trabecular bone, like lumbar spine (2, 13), with significant lower BMD values related to disease duration and regardless of treatment (14). A reduction in BMD also characterizes periarticular local bone loss in RA (15, 16), and this manifestation seems to be associated with the development of aggressive systemic disease (17). Glucocorticoids (GC) are often prescribed at a higher dose in the treatment of RA and its detrimental effect on the bone with increased risk of fragility fracture has long been documented in the literature (3). Dual X-ray absorptiometry is widely used also in rheumatological diseases to assess BMD (18), but the occurrence of fragility fractures in GC patients at higher than expected BMD, with a risk factor substantially independent of BMD, arises the question if other bone factors than density have to be assessed for a better comprehension of bone failure (19). Trabecular bone score, an indirect DXA index of bone texture, appears to be a valid index of bone quality that may explain fracture events at a higher BMD in patients receiving GC (3, 20). However, TBS, as a lumbar spine textural index, does not provide all the necessary information to evaluate the resistance of bone to compressive, torsional e flexural loads. Hip geometry is another constructive data that could be of help in understanding bone failure. Hip structural analysis derived from a DXA femoral scan provides useful parameters to assess bone resistance to flexural and torsional loads, like those acting on the femoral neck (21). Despite its premises and promises to data, there is no clear evidence that HSA is useful in RA bone assessment.

The bone quality assessment has also been considered in other rheumatological diseases, like Systemic Lupus Erythematosus (SLE), Systemic Sclerosis (SS), Ankylosing Spondylitis (AS), in order to improve fracture risk assessment. Degraded bone texture measured by TBS appears to be associated with a prevalent vertebral fracture in SLE (22). Patients with SLE also show a derangement in bone geometry, with correlation between major/hip fractures, SLE duration, steroid use and neck buckling ratio (BR), index of neck stability under axial loads, in a long follow up cohort of patients (23). Trabecular bone score has been investigated in SS with a finding of a correlation with a condition of more altered microvascular damage (24, 25). In AS patients, lower TBS is associated with vertebral fractures (26) and the severity of disease in young male (27).

Bone mineral density, TBS and HSA are undoubtedly useful, particularly BMD, to assess bone status in rheumatological disease, but from a constructive point of view, they provide incomplete information about bone resistance to load, whereas strength relating data are missing. A new DXA-derived index based on the Finite Element Analysis (FEA) on a greyscale of density distribution measured on spine and femoral scan, namely Bone Strain Index (BSI), has recently been developed. Bone Strain Index includes local information on density distribution, bone geometry and loadings and it differs from BMD and also from other variables of bone quality like TBS, which are based on the quantification of bone mass and its distribution, averaged over the scanned region. Bone Strain Index appears to be a new frontier in the bone assessment that could provide useful information to a better comprehension of bone quality derangement in rheumatological diseases. This state of art review illustrates the methodology of BSI calculation, the findings of its in reproducibility and the preliminary data about its capability to predict fragility fracture and to monitor the follow up of the pharmacological treatment for osteoporosis. The text was structured in a chapter titled “Beyond Bone Mineral Density” with three sub-chapter: the first titled “Background” with the overview of the mathematic and the physic underlying the BMD derived indexes; the second titled TBS with the description of the TBS index; the third titled “Hip geometry” with the explanation of the hip structural analysis; the fourth titles “BSI” with the description of the new DXA index and the scientific evidence published in the literature. The Authors have consulted PubMed and Scopus. The literature considered for BSI was restricted to the *in vitro* and *in vivo* clinical studies. Search terms were: bone strain index, strength index of bone and their acronyms.

### Beyond Bone Mineral Density

#### Background

Bone is assimilable from the constructive point of view to a complex object, built with a particular design of its structure and geometry, in order to meet the mechanical and metabolic requirements that characterise its natural function. The mechanical function is primarily that of the resistance of the skeleton to loads, both compressive, torsional and flexural.

When a structure is loaded, stresses and strains are generated inside the object. The distribution of these stresses, their magnitudes and their orientations throughout the structure, depend not only on the loading configuration, but also on the geometry of the structure and of the material properties. The object is preserved until these stresses and strains remain below a certain level of solicitation named yield point, above which permanent damage starts to occur, until final fracture. Thus, despite the widespread belief in non-engineering environments, bone resistance is governed by several mechanical parameters that relate to bone density, bone geometry, internal trabecular structure and cortical thickness. Investigation and definition of these parameters are typically based on medical images, and particular attention is required to understand the appropriate mechanical meaning. Depending on the technology used to acquire the image, the quantification of these features can rely on 3D data, in case of computed tomography (CT) usage, or 2D data as traditional radiography (X-ray) and dual-energy X-ray absorptiometry (DXA).

In CT derived images, local bone material properties are typically defined by converting voxel values in Hounsfield unit (HU), to bone mineral density values (BMD) (ρ = a × HU + b). Geometrical aspects of the bone, like the shape and the cortical thickness, can be accurately measured in the whole bone volume. Furthermore, models based on 3D images can directly describe the architectural design for proper evaluation of trabecular structure quality.

On the other hand, the amount of data and measures acquired in 3D, leads to high level of complexity in the analysis, that require thorough mechanical knowledge and a more in-depth evaluation in the clinical process. Several methods have been proposed in order to take into account the different aspects involved in the resistance evaluation of an object (28–30).

Classic (Euler–Bernoulli) beam theory provides a calculation method to assess the level of solicitation of a beam and has been extensively applied for stress estimation in long bones (28, 31, 32). Although methodologically straightforward, it requires the objects to be approximated as beams, sometimes resulting in an oversimplification of the real situation, especially for complex irregular bones.

Another method used in engineering is a mathematical approach called Finite Element Model (FEM) (33). The FEM concept is based on the idea that a complex problem can be divided into simpler and smaller elements that easily can be handled to find the solution. In particular, the Finite Element Model applied to structural simulation requires the definition of the object by a simple shape element mesh, the definition of the material properties and the definition of boundary conditions (constraints and forces acting on a system). As a result, stress and strain distribution inside the considered object can be evaluated for proper fracture risk assessment.

Finite Element Model has been applied for many years in the design and manufacturing processes, and still today represents the standard reference for many engineering applications. In the medical field, it was introduced first to orthopedic biomechanics in 1972 (34). Since then, it has been applied with increasing frequency, and it is still used today. In recent years several implementations of models based on CT images have been proposed (35–37).

Most of the studies were successful in showing that FEA strength predictions outperform areal BMD as a predictor for fracture for their respective datasets (8). Although the use of Finite Element Method represents a simplification of reality, the “computational cost” associated with 3D models is still too high and, to date, cannot be included in the routine clinical evaluation.

Furthermore, QCT-based finite elements models demonstrated to be extremely sensitive to the different acquisition protocols and model definitions. The positioning of the patient, the slice thickness, the field of view (FOV) and reconstruction kernel (an algorithm that filters the acquired images before reconstruction in CT acquisition), inter-scanner variation, and manual definition of boundary conditions are just some of the several parameters that could lead to errors that may vary from 4% to up to 20% (38, 39). Further aspects that limit the spreading of CT based FEM in the clinical practice for fracture risk evaluation purposes are the low availability of high-resolution CT, and major invasiveness of the examination compared to DXA (40).

2D Models are a further simplification of the reality that in the last decade has been investigated with different assumptions (41–44). Even though they all rely on DXA images or simulated DXA images, differences in the implementation and design aspects may influence results and comparisons.

Luo et al. first introduced three fracture risk indices expressed as ratios of internal forces caused by impact forces occurring in sideway fall to bone ultimate cross-section strength at the femoral neck, intertrochanteric region, and subtrochanteric region (41, 45). The proposed finite element modeling procedure was validated against six representative clinical cases, where initial and follow-up DXA images have been taken to monitor the longitudinal variation of areal BMD. It was found from the clinical validation that variations in the proposed fracture risk indices have the same trends as those indicated by the conventional areal BMD and T-score.

In the same year, Den Buijs and Dragomir-Daescu validated a two-dimensional finite element method against experimental measurements with stress test and high-speed video recording (42). In this study, femur images were derived from the projection of quantitative computed tomography scans of human cadaveric femurs, and simulated FEM results were compared with the femoral stiffness and fracture load measured. Furthermore, digital image correlation analysis was used to calculate the strain distribution from the high-speed video recordings.

Later in 2013, Naylor et al. conducted a study to investigate whether bone strength derived from FEM analysis was associated with hip fracture risk in a longitudinal study. It was found that the DXA-based FE model was able to discriminate incident hip fracture cases from controls independently from FN BMD, prior fracture, VFA, and FRAX (44).

The association of estimated strength with incident hip fracture was partially confirmed in a subsequent case-cohort study by Yang et al. finding a correlation significantly better than Total Hip BMD and FRAX, but not significantly better than FN BMD (46). In this study for each DXA image was generated a stress ratio map (von Mises stress divided by the apparent yield stress), and the estimated femoral strength was calculated by scaling the peak impact force by the minimum stress ratio in the area with the highest stress.

Although several models have been developed in the last years, and although they all agree with underlying good prediction performance vs. Bone mineral density, there is still no consensus regarding which model best can describe the mechanical behaviour of bone. Significant differences can be found in material properties assignment, loading configurations and failure criteria.

In 2016 Dall'Ara et al. conducted a study to validate DXA-based finite element models to predict femoral strength in two loading configurations [one-legged standing configuration and side fall onto the greater trochanter (47)]. In both configurations, the DXA-based FE model provided a good agreement with the experimental data and demonstrated to predict femoral strength.

In a following *in vivo* validation study, Luo et al. found that automated FE model and femoral BMD could be applied to discriminate the fracture cases from the controls with considerably improved accuracy (48).

Recently, Yang et al. and Leslie et al., proposed femoral neck (FN), intertrochanter (IT), and subtrochanteric (ST) Fracture Risk Indices (FRIs) (45, 48), based on a plane stress model and the sideways fall assumption. In this case, bone failure was determined by the ratio between von Mises stress and bone yield stress over the defined ROI (e.g., femoral neck, intertrochanter, and subtrochanteric). Even though the coefficients of variation founded for the femoral neck, intertrochanter, and subtrochanteric FRIs were 5.5, 5.8, and 8.4%, respectively, the indexes were able to further stratify risk independently from BMD and FRAX, suggesting that they could improve potentially hip fracture risk assessment.

Although many FEMs have been developed for this purpose, most of them are not routinely used in the clinic. The main reason is that the computer programs that implement FEMs have not been completely automated, and heavy training should be required before clinicians can effectively use them.

Moreover, a standard tool should be available to investigate the mechanical behaviour of both femoral and lumbar anatomic site, usually scanned with DXA examination. Despite the availability of a certain number of studies investigating FEMs performance on the femur, only a few are dedicated to models of the lumbar spine.

A single two dimensional FEM of the first to fourth lumbar vertebra was first proposed by MacNeil et al. (43). In that model, bone tissue stiffness was assigned based on the BMD of the individual vertebrae, and adjusted for patient's age. Vertebral width was not measured from the image, but assumed to be constant for L1–L4 based on the height of L1 multiplied by 1.25, and middle vertebral width was assumed to be 95% of superior and inferior vertebral width.

Axial compression boundary conditions were applied with a force proportional to body mass.

The FEM ROC curve of the overall strain demonstrated better performance compared to BMD.

Another study by Lu et al. demonstrated that the simulated DXA-based 2D FE model has a better capability for predicting the vertebral failure than densitometric measurements. Since there is currently no consensus on which failure criterion should be used for bone tissues, four different failure criteria were considered in this study: the principal stress, the principal strain, the von Mises stress and the equivalent strain. Yield stresses, Young's modulus, tensile and compressive yield strains were derived using empirical linear equations (49). One of the major outcomes of this study was that the failure loads predicted by the DXA-based 2D FE models using different failure criteria are strongly correlated with each other, demonstrating that adoption of different failure criteria has a minimal influence on the results of the 2D FEMs (50).

Another recent approach considers using bi-planar dual-energy (BP2E) X-rays absorptiometry to build vertebral FEM using sagittal and frontal plane radiographs from QCT scans (51). Compressive tests were conducted using uniform load application onto the upper surface of the specimen. Experimental vertebral strength was defined as the ultimate load achieved and axial stiffness was calculated as the slope of the linear region of the force-displacement curve. The results of this study suggest that FEMs are better experimental vertebral strength predictors than areal BMD measured with DXA.

In conclusion, although different assumptions may be used, any new FEM that use specific parameters for bone material properties, specific boundary conditions or failure criteria should be verified. The assumptions used to build the model may reflect reality in different degrees, and thus it is important to validate each model to determine its capability to predict a known outcome (52).

In the last years, a new parameter based on FEM and named BSI has been introduced (53). The Authors demonstrated a good correlation between yield strain index (calculated by using the load causing the yield in each sample) and experimental yield measured on porcine vertebra samples. As the average strain index calculated in the cited paper is closer to ultimate strength (${\text{R}}_{\text{adj}}^{2}$ = 0.65), the algorithm has then been adapted to human vertebrae, assuming a specific thickness of the model-dependent from the average width of the vertebra, from the material properties derived according to experimental Morgan equations (54), and from patient-specific loading configuration based on Han et al. study (55).

In the next sections of this work, we will investigate the basic concepts of DXA images and some of the derived indexes most used in clinical practice that demonstrated to add useful information to BMD. Dual X-ray absorptiometry is the gold standard method in clinical practice to assess and to monitor bone status, due to its high accuracy, widespread of bone densitometry, low cost and low radiation exposure (56). The measured BMD is an areal BMD (g/cm^2^) and therefore differs in technical terms from the physical definition of density of an object (g/cm^3)^, and thus also from the volumetric BMD measured by CT.

Being DXA a projective X-ray device, the BMD measured in a district is a function of bone mineralisation (and therefore of volumetric BMD), and the amount of bone encountered in the X-ray direction, which in turn is related to the thickness (32).

**Figure 1:**
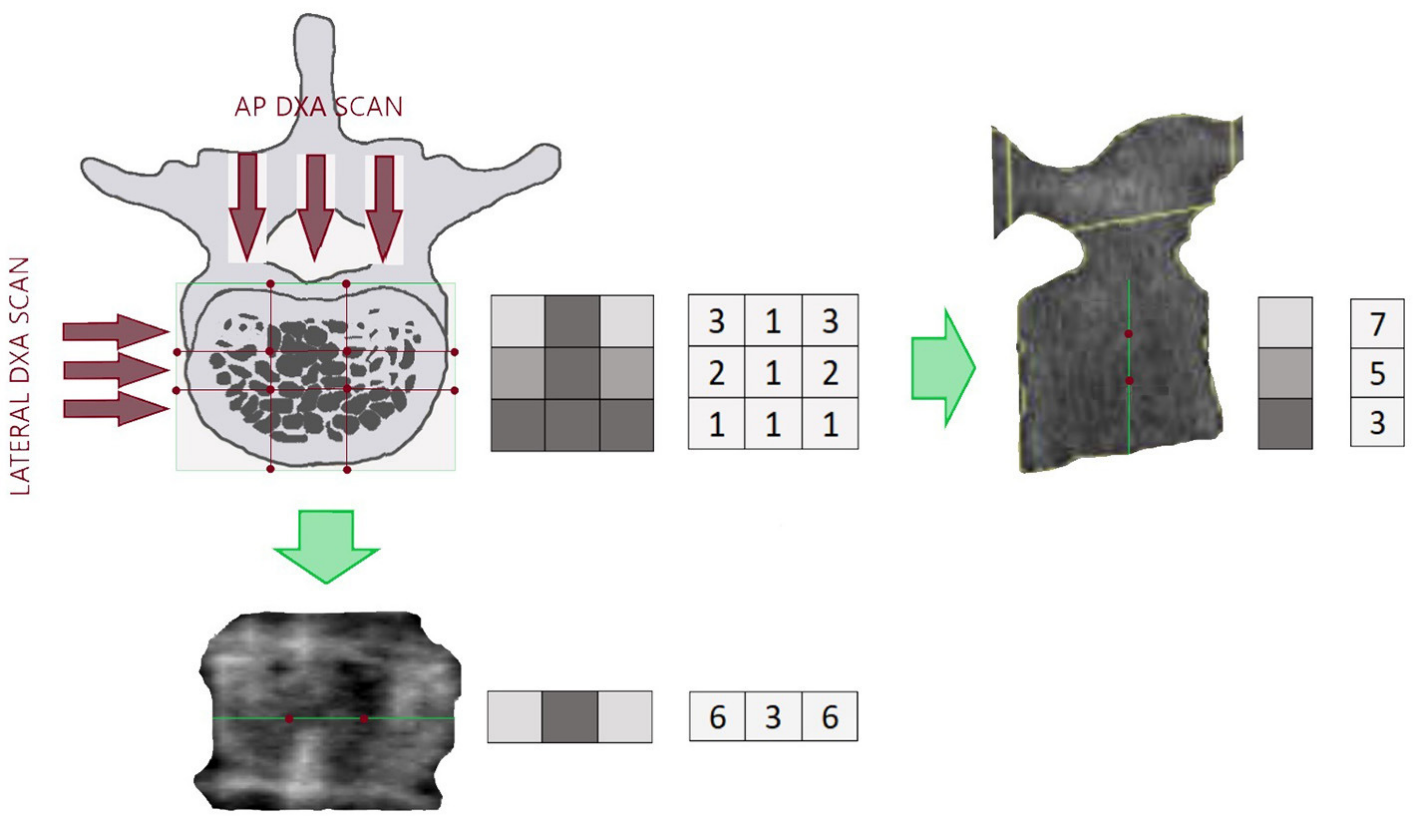
Example of a section of a lumbar vertebra with the relating lateral DXA scan (right) and the Antero Posterior (AP) DXA scan (bottom). The green lines on the DXA images represent the projection of the lumbar section in the X-ray direction. Red points are reference points that separate areas with different density. On the right side of each picture there is a representation of BMD distribution in the investigated section and the resulting lines, being the lines the projection of the lumbar section. Density distribution is shown in a grayscale form and numerical form, to better understand how high density (light gray colored squared) and porosity (dark gray colored squared) affect the resulting images.

As described in Figure 1, areal BMD can be represented by the sum of the mineral content in the X-ray direction. Bone mineral density is used in clinical routine to classify the patient in different risk classes depending on an epidemiological criterion of distribution of BMD in healthy subjects and patients affected by fragility fracture (57). Bone mineral density is also used to evaluate patient's response the pharmacological treatment prescribed to reduce fracture risk. However, assessment of BMD does not entirely explain fracture risk, since many patient fractures still occur in a population with normal or slightly reduced bone mass (58). Many other building factors of the skeleton have to be considered to explain bone strength (59) and to improve our ability to predict structural failure. Bone mineral density provides a valuable measure of the quantity of material used in the construction, but the architecture and external loads should equally be investigated to understand if the construction is appropriately designed.

#### Trabecular Bone Score

The TBS is a densitometric index that can be provided automatically analysing a lumbar DXA scan. Trabecular bone score evaluates bone mineral variations in lumbar DXA images in order to describe the internal structure of the bone.

Trabecular bone score calculation is based on the fact that DXA image areas with soft gray variations are typical of a dense trabecular structure. Conversely, big dark areas are characteristic of low connectivity, low trabecular number and wide space between trabeculae (60).

Referring to Figure 1 and keeping the same calculation method provided by Hans et al. (60) as an example, we can resume BMD and TBS different calculation as follows:

The DXA BMD resulting from the projection of the investigated lumbar section is given by summing in column (x-ray direction) the corresponding volumetric BMD

$$\begin{array}{l} 3+2+1=6\\ 1+1+1=3\\ 3+2+1=6 \end{array}$$

The BMD contribution of that line to areal BMD provided by DXA is 6 + 3 + 6 = 15 and does not depend on bone distribution inside the section.

Conversely, TBS calculation of that line is (3–6)^2^ + (6–3)^2^ = 18, and as explained in detail by Hans et al. (60), it does depend on the distribution of bone inside the section. In this example, it demonstrates how TBS value depends on the variation of density in the three different areas in the DXA line.

Of course, being TBS based on DXA image, it is able to explain porosity and density variation in the frontal plane, but it is not able to catch porosity and density variations in the sagittal plane, that for example should be visible from a lateral DXA scan (Figure 1).

The calculation of TBS is based on the same mathematical matrix DXA source used for BMD measurement, but it represents a different feature of bone status and is able to discriminate between patients with similar BMD, but different trabecular microarchitecture.

Trabecular bone score can discriminate fractured patients and can predict fracture, partially independently from BMD (61). More recently, the literature demonstrates that TBS is also useful in the follow-up of the pharmacological treatment of osteoporosis (62). Trabecular bone score usage has also been investigated in rheumatoid arthritis (63) showing its ability to detect patients with vertebral fractures in osteopenic population.

Recently, TBS behaviour has been investigated on DXA knee images to examine the bone quality at the distal femur and proximal tibia regions in patients with Spinal Cord Injury (64). Even though the software has been designed for the lumbar spine, in this study the L1, L2, L3, and L4 areas have been used to identify the diaphysis, the metaphysis and the epiphysis regions. The results indicate significant differences in TBS between groups only at femoral regions, despite large reductions in BMD at both distal femur and proximal tibia.

Another recent study that evaluates TBS performance on the distal femur and proximal tibia regions has been performed on patients that underwent Total Knee Arthroplasty (65). A TBS texture Research Investigational Platform (TRIP) that allows assessment of many skeletal sites has been used on DICOM images, observing lower values in the surgical leg, consistent with the bone loss that follows TKA (65).

Further research will be necessary to determine if TBS measurement at the knee, or other regions, may complement and strengthen fracture risk assessment.

#### Hip Geometry

**Figure 2:**
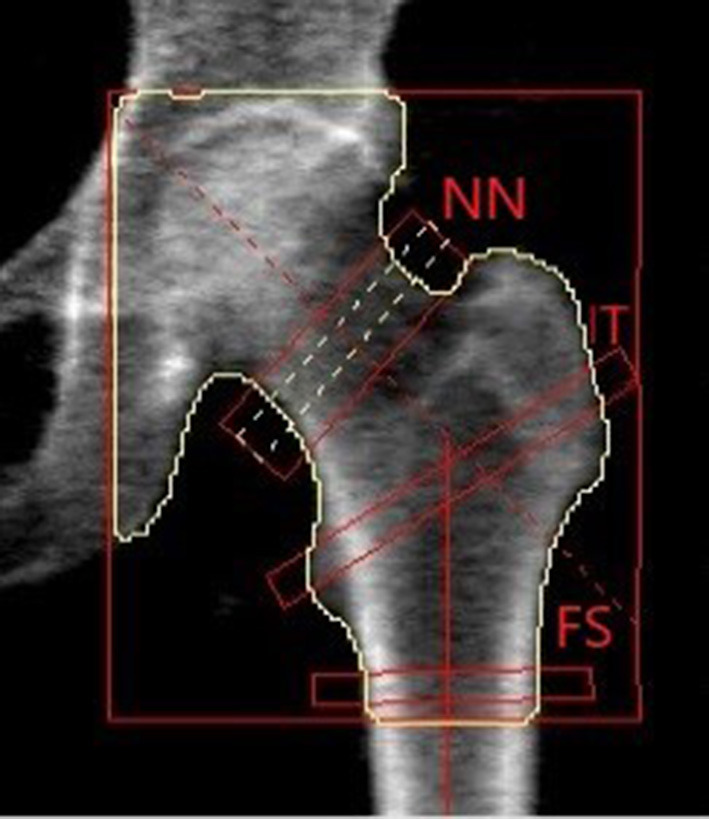
Hip image from a Hologic DXA scanner showing positions of thin analysis regions across the femur at the neck (NN region), intertrochanteric (IT), and shaft (FS).

Despite the predominant role that material properties, and thus BMD, had in fracture risk assessment, geometry and size are fundamental parameters that rule the mechanical resistance of the bone (66). In the last years, HSA programme has been proposed to provide a structural description of the proximal femur, and further improvement of fracture prediction (32). This method uses a proximal femur DXA image to extract information about cross-sectional geometry in three different regions of interest: the narrowest portion of the femur neck: narrow neck (NN), the inter-trochanteric region (IT) and the femoral shaft region (FS). For each location, the distribution of the bone mass is computed, and femoral mechanical properties are derived from femur geometry (Figure 2).

Hip structural analysis parameters are Cross-Sectional Area (CSA), indicative of the bone surface area in the cross-section; Cross-Sectional Moment of Inertia (CSMI), that describes how the bone mass is distributed around the femoral axis; Section modulus (Z), that indicates the maximum bending stress.

Under compression condition, forces parallel to the long axis are uniformly distributed over the surface of the cross-section (CSA). Conversely, considering bending loads, the resistance of bone varies at the square of the distance from the neutral axis. Thus, bone near the outer surface contributes to bone strength much more than bone near the femoral axis.

Considering that the femoral internal stresses and strains generated by external forces are mainly due to compression and bending (67), the higher are CSA and CSMI, the better is bone resistance, respectively, to axial compression and bending.

Another critical parameter provided by HSA is the ratio of the outer radius to the cortical thickness, named buckling ratio (BR). In engineering, buckling indicates a sudden collapse of an object subjected to an axial load, before the axial compression stress reaches the stress limit. If the ratio exceeds a factor of about 10, the cross-section begins to lose strength through susceptibility to local instability (32).

Studies showed that HSA results are important in predicting the occurrence of hip fracture (68, 69). However, the usage in clinical practice is still limited by the problematic interpretation of the several structural parameters associated with measured BMD and lack of evidence in clinical practice regarding fracture prediction (5).

Also International Society for Clinical Densitometry (ISCD) guidelines recommends that at this stage HSA parameters should not be used to assess hip fracture risk (70). Conversely, it has been reported in this official positions that Hip Axis Length could be clinically useful, being significantly associated with a fracture in various populations.

Another geometric parameter that can be automatically obtained by DXA images is the Neck shaft angle, but it is not yet clear if it can be used in clinical practice as a fracture risk parameter independent from BMD (70).

#### A New Index of Bone Strength: The Bone Strain Index

Over the past few years, FEM based on DXA images had particular prominence (30, 46). Considerable effort has been directed toward a variety of patient-specific structural engineering and FEMs of the proximal femur to estimate femoral strength and to assess hip fracture risk. However, just a few studies deal with the lumbar anatomic site. Recently, a new bone FEM structural DXA parameter named BSI, has been proposed in order to improve fracture risk prediction and take into account all the features involved in bone strength (53). Bone Strain Index calculation can be obtained in <10 s (=10 second) directly from images generated by DXA device (71). The automatic FEA uses a constant strain triangular mesh following the contour of the bone segmented by DXA dedicated software. The derived image is analysed building a separate model for each vertebra with the load applied to the upper plate and the constraints to the lower plate, according to the model described by Colombo et al. (53).

The thickness of the plane stress model is calculated from the average width of the vertebra, and material properties are assigned to each element according to the experimental formula provided by Morgan et al. at the lumbar site (54). The force acting on the upper plate is derived from simulated forces in standing position and for patient-specific weight and height (55).

Regarding the femoral region, BSI is calculated on the hypothesis of a sideway fall condition with constraints placed on the head and on the lower part of the shaft, and a force applied to the greater trochanteric area, according to Terzini et al. (72).

The thickness of the model is derived from the width of the central area of the neck region accordingly to previous studies (44, 46). Even for this anatomical site, the relations used to calculate the stiffness of each element of the model have been derived from the experimental equations in Morgan et al. study (54).

In both cases (lumbar and femoral), BSI represents the average equivalent strain inside the bone, with the assumption that a higher strain level (high BSI) indicates a more significant risk condition. Differently from the calculations provided in previous FEA studies regarding femoral region (44, 46, 72), the calculated BSI is not related to a specific strain/strength limit, according to the last studies focused on the lumbar region (73). No strain limit has been considered in the calculation of both parameters since specific strain distributions and limits should be found for different kind of populations depending on sex, age and pathology (52, 74, 75). Furthermore, yield point and stress-strain limits, in case of bone, are dependent on the material and on mineral composition (49, 76).

Being BSI a strain, this parameter is unitless because it refers to the variation of the size related to the original size (ε = dl/l_o_, where dl is the change of length and l_o_ is the initial length), and thus normally is expressed in percentage.

For easy reading, the average strain in the lumbar area has been expressed in percentage and multiplied by 100, whereas femur bone strain values must be intended as the percentage multiplied by 10. This order of size difference is expected because, accordingly to the models adopted so far, lumbar simulation is conducted in stand-up condition, whereas femur simulation is conducted in sideways fall, with higher forces acting on the bone.

The information provided by DXA through BMD, TBS and BSI describes the same situation from a different point of view. To understand what each tool provides to the clinician, one has to consider a parallelism with two horizontal structures that must support the weight of two different persons.

**Figure 3:**
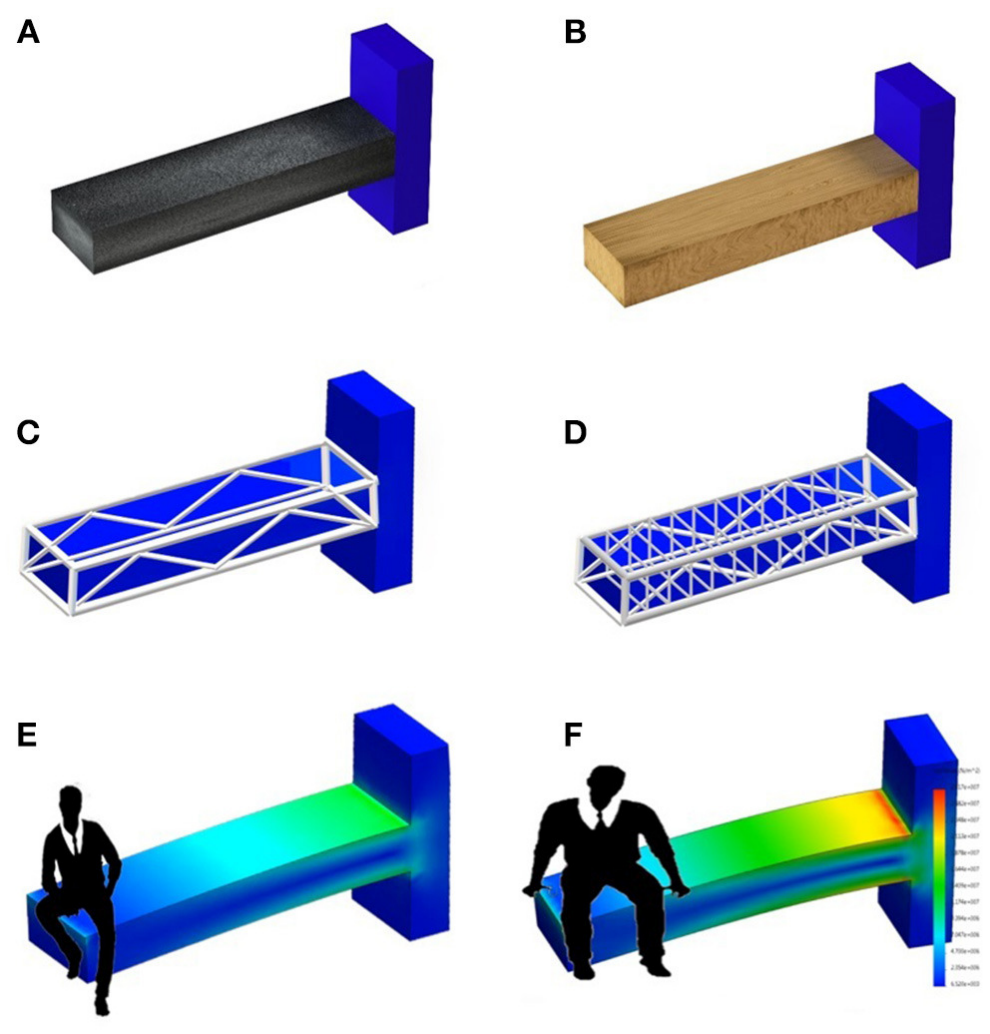
Example of the information level provided by BMD, TBS and BSI related to a man sitting on a beam. **(A,B)** Show the difference in materials (e.g., concrete and wood) that can be assessed by BMD DXA measurement. **(C,D)** Relate to internal structure design showing a difference between a dense and a sparse structure, and using the same concept of TBS. **(E,F)** Show the stress-strain status of a bar made up of a specific material and with a specific structure, with two different people sitting on the top. The information provided by **(E,F)** is the same provided by BSI.

As shown in Figure 3, BMD describes the material (e.g., wood or concrete), whereas TBS is an independent parameter that illustrates the different inner structure of the two beams. In both cases, detailed geometry information and load definition are missing. To access a complete view of the situation, we should take into account all the above variables to evaluate the proper mechanical behavior and the risk of failure.

Bone Strain Index calculation is obtained using a constant strain triangular mesh following the contour of the bone segmented by DXA software. Bone Strain Index model relies on a triangular mesh built around the bone segmented in DXA software. Each triangle of the model has an Elastic Modulus depending on the BMD of the object, according to Morgan et al. equations (54).

The force acting on the object is precisely calculated and based on each patient's height and weight. Furthermore, the distribution of the strain is represented on the object showing the location of the most stressed region. If BMD can be defined as a bone quantity value and the TBS as a bone quality value, BSI should be described as a bone strength value, being its nature related to the capability to withstand an applied load.

**Figure 4:**
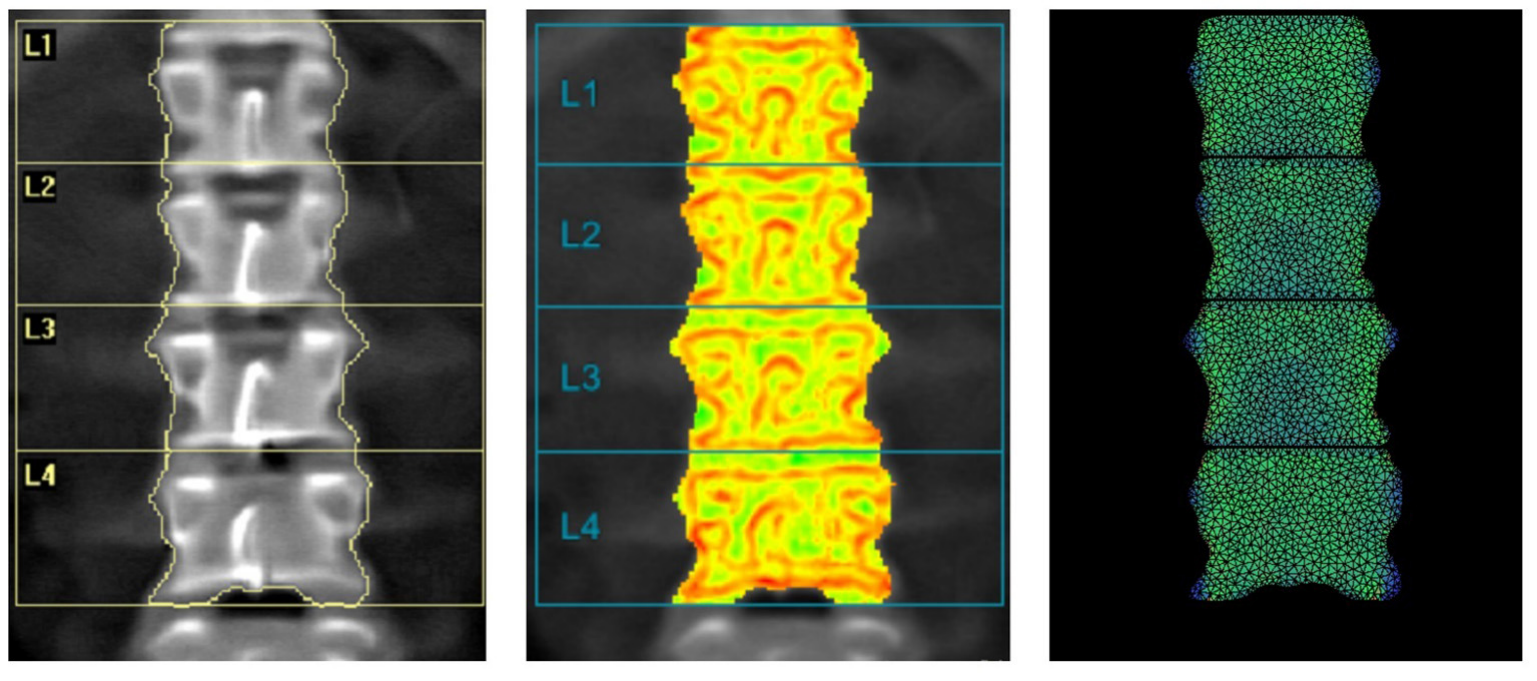
Example of images provided by DXA: BMD L1L4 = 0.868 g/cm^2^ (left); TBS L1-L4 = 1.361 (center) and BSI L1L4 = 1.57 (right).

Figure 4 shows a comparison between DXA image, TBS image and BSI image with a superimposed triangular mesh. Trabecular bone score image shows in red the areas with low TBS values and in green the areas with high TBS values, where TBS value is based on the variations of gray level related to the trabecular structure, as previously explained.

**Figure 5:**
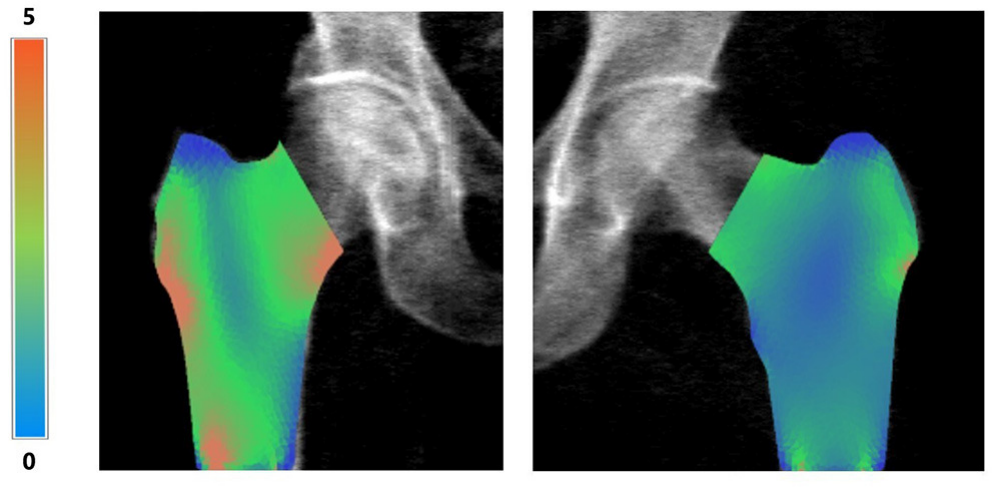
Examples of right femur BSI analysis (on the left, BMD total value = 0.55 g/cm^2^, Femur BSI = 2.09) and of left femur BSI analysis (on the right, BMD total value = 0.82 g/cm^2^, Femur BSI = 1.29) of two different patients. The heat map related to the strain distribution shows a major strain concentration on the red area. The head of the femur is not represented because the colored regions represent the same regions identified by DXA analysis (neck, intertrochanteric and trochanteric).

Bone Strain Index image, conversely, represents the strain distribution inside the object with a colour scale that goes from blue/green (low strain) to yellow (mid strain level) and red (high strain), as shown in Figure 5.

Since trabecular distribution in the femur region is asymmetric and more complicated, no TBS-like evaluation has yet been developed for femoral trabecular structure. Conversely, BSI evaluation for femoral region follows the same criteria used for the lumbar region, except for material properties and boundary conditions.

An example of BSI calculation of right and left femur is presented in Figure 5 without the superimposed mesh.

Recent clinical studies have investigated the usefulness of BSI to identify the osteoporotic patient's subgroup particularly prone to fragility fractures (77) and to predict further fragility fractures (73, 78) (Table 1). Ulivieri et al., using artificial neural network analysis (ANNs), investigated 125 consecutive postmenopausal women assessing bone quantity and quality DXA parameters, biochemical markers of bone turnover and clinical data. A low fracture risk seemed to be related to a low carboxy-terminal cross-linking telopeptide of type I collagen level, whereas a positive Romberg test, together with compromised bone strength DXA parameters (high lumbar BSI), appears to be strictly connected with fragility fractures, indicating in this way the path that leads to fragility fracture in a postmenopausal population (77). More recently, Messina et al. have demonstrated in a multicentric validation study, that lumbar BSI is an independent predictor of a subsequent fragility re-fracture (78). The Authors investigated 234 consecutive fractured patients with primary osteoporosis who performed a spine X-ray for the calculation of Spine Deformity Index (SDI) and DXA densitometry for BMD, TBS and lumbar BSI measurement at the basal time and in the follow up at each clinical check. A subsequent fracture has been considered as one unit increase of SDI. For each unit increase of the investigated indexes, the univariate hazard ratio of re-fracture, 95% CI, *p*-value and proportionality test *p*-value are: for age 1.040; 1.017–1.064; 0.0007; 0.2529, respectively, and for lumbar BSI 1.372; 1.038–1.813; 0.0261; 0.5179, respectively. Lumbar BSI remained in the final multivariate model as a statistically independent predictor of a subsequent re-fracture (1.332; 1.013–1.752; 0.0399) together with age (1.039; 1.016–1.064; 0.0009. Multivariate model proportionality test *p*-value was 0.4604.

**Table 1:** Bone Strain Index reproducibility and clinical studies.

**Topic**	**Author**	**Year**	**Patients no**.	**Main findings**
*In vivo* reproducibility	Messina et al.	2020	30	BSI *in vivo* reproducibility of Total Femur (CoV = 3.89%, reproducibility = 89.22%) was better compared to that of Femur Neck (CoV = 4.17%, reproducibility = 88.46%).
Prediction of vertebral re-fracture (multicentric retrospective study)	Messina et al.	2020	234	BSI hazard ratio (95% CI) of incident re-fracture for each unit increase was 1.372 (1.038–1.813), *p-*value = 0.0261, proportionality test *p-*value: 0.5179.
Bone geometry and structural indexes in Mastocytosis (retrospective study)	Ulivieri et al.	2020	96	Tryptase showed a statistically inverse correlation with Lumbar Spine BMD (*r* = −0.2326; *p* = 0.0226) and with TBS (*r* = −0.2801; *p* = 0.0057) and a direct correlation with Lumbar BSI (*r* = 0.2759; *p* = 0.0065). In the multivariate regression model only the Lumbar BSI remained statistically significant in systemic Mastocytosis (*p* = 0.0064) and non-systemic Mastocytosis (*p* = 0.0338).
Prediction of vertebral re-fracture (multicentric retrospective study)	Ulivieri et al.	2020	143	The hazard ratio of re-fracture for each unit increase of BSI, BMD and TBS were, respectively, 1.201, 0.231, and 0.034. BSI resulted in being the nearest to the statistical significance to predict a re-fracture, with greater values associated with higher re-fracture risk.
DXA parameters response to Teriparatide (retrospective study)	Messina et al.	2020	40	In the entire population, the ameliorations after therapy regarded BSI (-13.9%), TBS (5.08%), BMD (8.36%). Significant HSA variations were shown only at the femoral shaft, but of very small entity [FS_BMD (0.23%), FS_CSA (−0.98%), FS_SEC_MOD (−2.33%) and FS_BR (1.62%)].
*In vivo* reproducibility	Messina et al.	2020	150	BSI best reproducibility value was observed in the group with BMI between 25 and 30 kg/m^2^ (CoV 1.97%, reproducibility 94.5%), while the worst was in the group with BMI > 30 kg/m^2^ (CoV 3.96%, reproducibility 89.0%). BSI reproducibility progressively worsened from lower BMI to higher BMI, but the amount of this reduction was never statistically significant.
*In vitro* reproducibility and soft tissue thickness influence	Messina et al.	2019	Phantom based study	The highest value of BSI reproducibility was 98.3% (1-cm soft tissue thickness, HD-mode), whereas the lowest one was 96.1% (6 cm soft tissue thickness, HD-mode). Variations between scans with superimposed 0–6 cm thickness of soft tissue were between 0.76% and 1.46% for BMD, and between 1.03% and 1.57% for BSI.
DXA derived parameters in haemophilic patients (retrospective study)	Ulivieri et al.	2018	70	A reduced bone mass was present at the femoral neck in 55.7%, at total femur in 18.6%and at the lumbar spine in 54.3% of patients. Lumbar spine BMD, TBS and lumbar BSI did not correlate with HJHS (Hemophilia Joint Health Score). HSA bone geometric parameters correlated negatively with HJHS.
Clinical observational retrospective study	Ulivieri et al.	2018	125	A low fracture risk seems to be related to a low carboxy-terminal cross-linking telopeptide of type I collagen level. In contrast, a positive Romberg test, together with compromised BSI, appears to be strictly connected with fragility fractures characterizing the pathway leading to fracture in postmenopausal women.

Lumbar BSI also recently demonstrated its ability to characterise young patients affected by secondary osteoporosis (77, 79) (Table 1). As regard patients affected by mastocytosis the Authors found a relation between lumbar BSI and severity of bone deteriorating involved in 96 consecutive patients (46 women and 50 men) affected by cutaneous (CM) or systemic (SM) mastocytosis. Tryptase was inversely correlated with lumbar BMD (*r* = −0.232; *p* = 0.022) and TBS (*r* = −0.280; *p* = 0.005), and directly with lumbar BSI (*r* = 0.276; *p* = 0.006). Lumbar BSI remained statistically significant (*p* = 0.006; adjusted *R*^2^ = 0.101) in the multivariate regression model with Tryptase as dependent variable, being lumbar BMD and TBS not statistically significant. Tryptase increased about 22 units for each unit increase of lumbar BSI. Moreover, lumbar BSI was statistically lower in women than in men, suggesting that men have a worse lumbar bone resistance to compressive loads, according to the more severe bone involvement of mastocytosis in the male sex (79) (Table 1).

Another aspect that contributed to DXA conventionally established as the gold standard method for the diagnosis of reduced bone mass and its follow up is the higher reproducibility and precision (80). International Society for Clinical Densitometry states that precision is defined as the ratio between standard deviation and mean (CoV). Per cent least significant change (LSC%) is calculated as 2.77 × CoV, and reproducibility is calculated as the complement to 100% of LSC% (7). Usually, BMD reproducibility is known to be very good and typically represents the standard of reference for other DXA-based measurements. This has been recently confirmed by Messina et al. BMD reproducibility ranging around 99% in all the densitometric scan modalities, while the reproducibility of BSI is lower than that of BMD being the CoV found between 0.6 and 1.4% and the LSC about three times higher than that of BMD (81, 82).

An overview of BMD and BSI *in vitro* and *in vivo* precision is reported in Table 1. For what concerns *in vivo* results, a comparison between different BMI groups and different waist circumference is reported in Messina et al. study, where almost the same difference between BMD and BSI reproducibility has been found on the previous phantom study (83). Worse reproducibility compared to BMD has been already investigated in other bone quality parameters (84, 85), suggesting that BMD measurement still represents the best choice for detecting small bone variation in the disease's follow up. Despite this, not necessarily BMD small variations result in significant changes in bone structure and bone strength, as indicated by the TBS and BSI LSC. Thus, these investigation tools maintain unaltered their ability to describe bone quality and bone strength status but require a longer period to observe significant variations.

Moreover, reproducibility of all DXA parameters (BMD, TBS and BSI) slightly worsen in obese patients and in those with the greater waist circumference. This behaviour can be commonly justified by the soft tissue superimposed to the bone, that affects the x-ray image generating noise and reducing image quality and accuracy (86).

A recent clinical study validated the BSI ability to monitor the effect of anabolic treatment for osteoporosis (87). Forty osteoporotic patients with fractures were studied before and after 2 years of daily subcutaneous 20 mcg of teriparatide and BMD, HSA, TBS, and BSI were measured. Both classical statistical approach and ANNs were used for the analysis that demonstrated a significant amelioration after therapy regarded BSI (−13.9%), TBS (5.08%), BMD (8.36%). Dividing patients into responders (BMD increase >10%) and non-responders, the first presented TBS and BSI ameliorations (11.87% and −25.46%, respectively, while non-responders presented an amelioration of BSI only (−6.57%). This finding suggests that an increase in bone strength may explain the known reduction in fracture risk not merely justified by BMD increase.

The limitation of all the reported clinical studies is the utilized samples size, if compared to the magnitude of the cases involved in BMD and TBS studies. However, it must be considered that BSI is a recently proposed index and the validation studies on large case series, of thousands of patients, require a long time, and they are still in progress.

#### The Frontiers of DXA in Rheumatology

Rheumatological diseases present bone involvement characterise not only by bone quantity but also by bone quality impairment. Osteoporosis secondary to rheumatological diseases is a multifactorial local and systemic pathology aggravated by the intake of glucocorticoids which represents one the more significant factor interfering with bone resistance to load and fatigue. The amount of bone, its spatial distribution, its geometry and its strength determine skeletal resistance to load and fatigue and a complete clinical assessment of fracture risk needs to identify and measure all these characteristics. Trabecular bone score, nowadays, is a widely studied bone textural index, able to discriminate fractured patients and to predict fracture both in primary and in secondary osteoporosis where bone architecture is damaged. Hip structural analysis needs further evidence of its ability in bone geometry assessment and fracture risk prediction.

Bone strength is the last field where knowledge is necessary to understand all the physical implications of bone resistance to loads and fatigue in order to provide the medical clinician with all what is necessary to manage better patients affected by rheumatological diseases. BSI appears to be a powerful index of the strength of the bone that will provide informations on the physics of the skeleton resistance to the loads that are still lacking.

## Author Contributions

FU and LR have conceptualized and written the manuscript. Both authors contributed to the article and approved the submitted version.

## Conflict of Interest

The engineer LR, former working in Politecnico of Turin and now employed at the commercial company TECHNOLOGIC S.r.l, has extracted and tabulated the densitometric data and has applied the mathematical algorithms based on the finite element analysis to calculate the Bone Strain Index. TECHNOLOGIC S.r.l. Provided support in the form of salary for LR, but did not have any role in the study design, data collection and analysis, decision to publish or preparation of the manuscript. The remaining author declares that the research was conducted in the absence of any commercial or financial relationships that could be construed as a potential conflict of interest.

## Abbreviations

DXA: dual X-ray absorptiometry
BMC: bone mineral content
BMD: bone mineral density
BSI: bone strain index
TBS: trabecular bone score
FEA: finite element analysis
FEM/s: finite element model/s
HAS: hip structure analysis
NN: narrow neck
IT: inter trochanter
FS: femur shaft
CSA: cross section area
CSMI: cross section moment of inertia
Z: section modulus
BR: buckling ratio
RA: rheumatoid arthritis
GC: glucocorticoid
SLE: systemic lupus erythematosus
SS: systemic sclerosis
AS: ankylosing spondylitis
HU: hounsfield unit
CT: computed tomography
BMI: body mass index.
